# Pansharpening of WorldView-2 Data via Graph Regularized Sparse Coding and Adaptive Coupled Dictionary

**DOI:** 10.3390/s21113586

**Published:** 2021-05-21

**Authors:** Wenqing Wang, Han Liu, Guo Xie

**Affiliations:** 1 School of Automation and Information Engineering, Xi’an University of Technology, Xi’an 710048, China; wangwenqing@xaut.edu.cn (W.W.); guoxie@xaut.edu.cn (G.X.); 2 Shannxi Key Laboratory of Complex System Control and Intelligent Information Processing, Xi’an University of Technology, Xi’an 710048, China

**Keywords:** pansharpening, multispectral image, panchromatic image, WorldView-2, graph regularized sparse coding, dictionary learning

## Abstract

The spectral mismatch between a multispectral (MS) image and its corresponding panchromatic (PAN) image affects the pansharpening quality, especially for WorldView-2 data. To handle this problem, a pansharpening method based on graph regularized sparse coding (GRSC) and adaptive coupled dictionary is proposed in this paper. Firstly, the pansharpening process is divided into three tasks according to the degree of correlation among the MS and PAN channels and the relative spectral response of WorldView-2 sensor. Then, for each task, the image patch set from the MS channels is clustered into several subsets, and the sparse representation of each subset is estimated through the GRSC algorithm. Besides, an adaptive coupled dictionary pair for each task is constructed to effectively represent the subsets. Finally, the high-resolution image subsets for each task are obtained by multiplying the estimated sparse coefficient matrix by the corresponding dictionary. A variety of experiments are conducted on the WorldView-2 data, and the experimental results demonstrate that the proposed method achieves better performance than the existing pansharpening algorithms in both subjective analysis and objective evaluation.

## 1. Introduction

For several decades, a huge amount of remote sensing images, which are provided by optical satellites, played a crucial role in human tasks. With an increasing demand for very high-resolution (HR) products, high-performance acquisition devices are quickly being developed. Nevertheless, due to physical constraints, a sole acquisition device cannot provide very fine spatial and spectral resolutions [1]. Normally, the optical satellites are equipped with two types of imaging devices: multispectral (MS) and panchromatic (PAN). The MS image is composed of several spectral channels and has rich color information. However, its spatial resolution does not satisfy the requirements of some remote sensing applications, such as classification and objection detection. The PAN image with only one spectral channel can supply high spatial resolution. Thus, pansharpening (PS) technique, which fuses the MS image and the PAN image, was developed to acquire HR MS images [2].

Nowadays, the existing PS approaches can be classified into three categories: component substitution (CS), multiresolution analysis (MRA), and variational optimization (VO)-based methods [3]. The CS-based methods, also known as spectral approaches, project the MS image onto a specific space and substitute the component that contains the main spatial information with the histogram-matched PAN image. This category of methods consists of intensity-hue-saturation (IHS) [4], Gram–Schmidt (GS) spectral sharpening [5], and principal component analysis (PCA) [6]. Due to the obvious spectral distortion caused by the classical CS-based methods, some improved methods belonging to this category were presented, which can be found in the literatures [7,8,9,10]. The MRA-based methods, also known as spatial approaches, constitute another category of PS approaches. This category injects the spatial details extracted from the PAN image through a multiresolution decomposition into the upsampled MS image of the same scale with the PAN image. Several modalities of MRA are used to extract the spatial details, such as decimated wavelet transform [11], undecimated wavelet transform [12], “à trous” wavelet transform (ATWT) [13,14], Laplacian pyramid (LP) [15], contourlet transform [16], curvelet transform [17], generalized LP based on Gaussian filters matching the modulation transfer function (MTF) of the MS sensor (MTF-GLP) [18,19,20], and so on. The MRA-based methods are well able to preserve the spectral information but may cause spatial distortions in local regions. Since these two categories of methods produce the fused images with different features, hybrid methods combining the advantages of CS-based and MRA-based methods were developed. The representative methods include IHS+Wavelet [21], PCA+Contourlet [6], and ICA+Curvelet or Wavelet [17,22], etc.

As a new generation of PS methods, the VO-based methods attracted much attention from researchers and were rapidly developed [3]. This category of PS methods generally coverts the pansharpening process to the optimization of a variational model. As a crucial part for these methods, the construction of energy functional relies on the imaging mechanism of the observed measurements [23,24,25]. The energy functionals generally consist of three parts, i.e., the spectral fidelity model, the spatial enhancement model, and the prior model [3]. The spectral fidelity model aims to preserve the color information of the MS image as much as possible. It describes the relationship between the ideal fused image and the low-resolution (LR) MS image. The LR MS image can be regarded as a degraded version of the ideal HR MS image processed by blurring, downsampling, and noise operators [25,26]. The spatial enhancement model constructs the relationship between the ideal fused image and the PAN image. Two main assumptions are usually made in this model: one assumption is that the PAN band is represented as a linear combination of the HR MS bands [26]; the other assumption is that the spatial structures of the pansharpened image are approximately consistent with the PAN image [27,28]. The prior model that imposes the spatial constraints on the ideal HR MS image is used to further enhance the spatial quality. The representative prior models include Laplacian prior [29], total variation [30], nonlocal prior [31], low-rank prior [32], etc. Based on the idea of image super-resolution, sparse representation (SR) technique was successfully used in remote sensing image fusion [33,34]. The SR-based method assumes that the LR images and the HR images have the same coefficients under certain specific dictionaries. During the last ten years, a lot of SR-based PS methods were proposed [35,36,37,38,39,40,41,42,43,44,45,46,47,48,49]. These methods adopt different dictionary construction approaches to improve the fusion performance. Although the SR-based method has better performance than the CS and MRA methods, its high computational complexity restricts practical applications.

With the rapid development of the deep learning techniques, various types of convolutional neural network (CNN) structures proven to be effective in classification and super-resolution tasks were applied to remote sensing image fusion [50]. The representative methods include Pansharpening CNN (PNN) [51], multiscale and multidepth CNN (MSDCNN) [52], PNN with a spectral-spatial structure loss (S3) [53], Pan-GAN [54], GTP-PNet [55], etc. These methods accomplish better results than the traditional PS methods. However, the CNN-based pansharpening methods require many datasets to train the network structures and have weak generalization ability for different types of remote sensing images.

**Figure 1:**
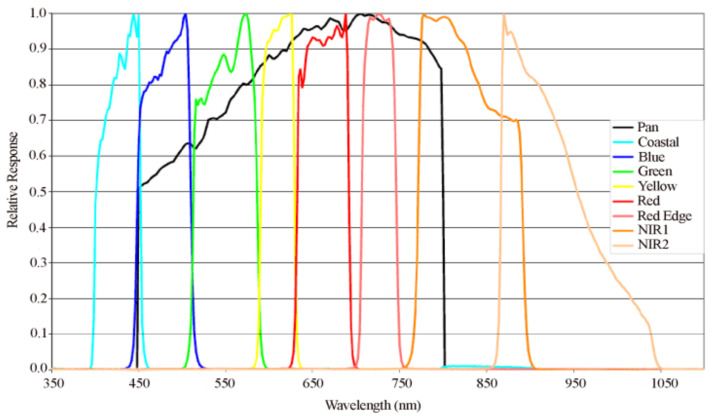
Relative spectral response of WorldView-2 data (colored lines for MS channels and black line for PAN channel).

However, for different types of PS methods, the spectral mismatch between the MS channels and the PAN channel can result in an unwarranted degradation of fusion performance. Figure 1 shows the relative spectral response curve of WorldView-2; the colored lines and black line indicate the spectral responses of the MS channels and the PAN channel, respectively. The yellow, red, and red edge bands are within the wavelength range well covered by the PAN band. Also, the blue and green bands are partially outside the wavelength range covered by the PAN image, while the coastal, NIR1, NIR2 bands are almost outside the wavelength range covered by the PAN image. Hence, an obvious difference exists in the spectral response for the WorldView-2 data. The spectral mismatch problem makes most of PS methods suffer from spectral and spatial distortions. For example, the VO-based methods usually adopt the linear combination model as the spatial enhancement term under the assumption that the spectral range of the PAN image almost covers that of all the MS channels. Hence, these methods are not suitable for pansharpening the WorldView-2 data. The sparse coding-based methods are based on the assumption that the LR and HR image patches have the same sparse representations over the dictionary pair learned from the PAN image and its degraded version. In our earlier work [56], we firstly exploited the graph regularized sparse coding (GRSC) [57] algorithm into the pansharpening. In this method, we only consider the four-band MS images; for the eight-band MS image, due to the spectral mismatch, the dictionary learned from the PAN image may not be adequate to sparsely represent the MS image patches. To reduce the influence of spectral mismatch, this paper proposes a PS method to sharpen the WorldView-2 data via graph regularized sparse coding and adaptive coupled dictionary (GRSC-ACD). Our contributions are as follows.

(1) Considering the degree of correlation among the MS channels and the PAN channel, the PS process of the WorldView-2 data is regarded as a multitask problem. The first task is to process the adjacent MS channels, i.e., green, yellow, red, and red edge, with high correlation to the PAN band and within the wavelength range well covered by the PAN image. The second task is to process a single MS channel, i.e., blue band, partially outside the wavelength range covered by the PAN image and with low correlation to the PAN image. The third task is to process the MS channels, i.e., coastal, NIR1, and NIR2 outside the wavelength range covered by the PAN image.
(2) To acquire precise sparse representations of the MS image patches, the GRSC algorithm is used in the GRSC-ACD method by exploiting the local manifold structure that describes the spatial similarity of the image patches. In each task, the LR MS channels are tiled into image patches, which make up an image patch set. Then, the image patch set is clustered into several subsets using the K-means algorithm so that the structural similarities of the image patches are further strengthened. Finally, each subset is sparsely represented by the GRSC algorithm. The accurate sparse representations contribute to a high-quality reconstruction of the HR MS image.
(3) Adaptive coupled dictionary is constructed for different PS tasks. For the first task, a coupled dictionary learned from the PAN image and its degraded version is used to sparsely represent the MS image patches. For the second task, to effectively represent the single blue band, the PAN image and the reconstructed green band that has high correlation to the blue band are selected as the image dataset to train the coupled dictionary. For the third PS task, the reconstructed blue band with high correlation to the coastal band is selected as the image dataset to learn the adaptive coupled dictionary for the coastal band. Meanwhile, the reconstructed red edge band is taken as the image dataset to learn the adaptive coupled dictionary for sharpening the NIR 1 and NIR 2 bands.

The rest of this article is organized as follows: Section 2 briefly introduces the SR-based PS methods, the SR theory, and the GRSC algorithm; the proposed GRSC-ACD method is presented in Section 3; Section 4 compares and analyzes the experimental results on degraded and real remote sensing data, and finally, Section 5 concludes this article.

## 2. Related Works

In this section, the background materials that our work is based on is briefly reviewed, including the SR-based PS methods, SR theory, and GRSC.

### 2.1. SR-Based PS Methods

During the last ten years, as an import branch of the VO-based methods, the SR theory made remarkable achievements in solving the PS problem. The first impressive work based on SR was proposed by Li et al., which assumes that the HR MS image patches have a sparse representation in a dictionary that is constructed by image patches randomly sampled from the HR MS images acquired by “comparable” sensors [33]. Although this method achieves superior performance compared to the aforementioned methods, the dictionary construction limits the applicability of this method because the ideal HR MS images are not available. To overcome this problem, several learning-based methods for dictionary construction were proposed [35,36,37,38]. In [34], Zhu and Bamler proposed SparseFI, a sparse coding-based PS method where a dictionary was learned from the PAN image and its LR version. This method opens up a new direction of PS, and it is based on the assumption that the LR patches and the HR patches share the same sparse representations. In [39], an extension of SparseFI, named J-SparseFI, was proposed by exploiting the possible signal structure correlations among the MS channels. To reduce spectral distortion, a two-step sparse coding method with patch normalization (PN-TSSC) was proposed [40]. In [41], a PS method featured with an online coupled dictionary learning was proposed, in which a superposition strategy was applied to construct the coupled dictionaries. Inspired by the MRA-based methods, Vicinanza et al. [42] proposed an SR-based PS method to estimate the missing details that were injected into the MS image by exploiting the details self-similarity through the scales. In [43], Tian et al. proposed a VO-based method based on gradient sparse representation. It assumes that the gradients of corresponding LR MS and HR PAN images share the similar sparse coefficients under certain specific dictionaries. Then, Tian et al. [44] proposed a VO-based PS method by exploiting cartoon-texture similarities, in which a reweighted total variation term using gradient sparsity is used to describe cartoon similarity and a group low-rank constraint is used to describe texture similarity. However, the SR-based PS method with patch manner suffers from two disadvantages: limited ability to preserve details and high sensitivity to misregistration. To overcome this problem, Fei et al. improved the above PS method by replacing the traditional SR model with a convolutional SR model [45]. Other similar PS methods were presented in [46,47,48,49]. These methods have good ability to preserve the spatial details and reduce the spectral distortion.

### 2.2. Sparse Representation

Recently, sparse representation became an effective technique for image processing applications [58]. It indicates that natural signals, such as images, are inherently sparse over the dictionary composed of certain appropriate bases. Let $x\in {ℜ}^{n}$ be a $\sqrt{n}\times \sqrt{n}$ image patch ordered lexicographically as a column vector. It can be represented as a linear sparse combination of basis atoms with respect to a dictionary $D\in {ℜ}^{n\times N}\left(n<N\right)$, which is defined as x=Dα, where $\alpha \in {ℜ}^{N\times 1}$ is the sparse coefficient vector with fewest nonzero elements. The sparsest α can be estimated through solving the following optimization problem:

(1) $$\underset{\alpha }{\arg\min}{\left\|\alpha \right\|}_{0}\;\mathrm{subject}\;\mathrm{to}\;{\left\|x-D\alpha \right\|}_{2}^{2}=0$$

where ${\left\|\cdot \right\|}_{0}$ is the $\ell_{0}$ norm that counts the nonzero elements in the sparse vector α, and ${\left\|\cdot \right\|}_{2}$ is the $\ell_{2}$ norm. However, the optimization problem in Equation (1) is nondeterministic polynomial-time hard (NP-hard). Hence, this optimization problem can be alternatively solved with the $\ell_{1}$ norm formulation, which can be represented as

(2) $$\underset{\alpha }{\arg\min}{\left\|\alpha \right\|}_{1}\;\mathrm{subject}\;\mathrm{to}\;{\left\|x-D\alpha \right\|}_{2}^{2}\leq \varepsilon$$

where ${\left\|\cdot \right\|}_{1}$ is the $\ell_{1}$ norm, and ε is the error tolerance. (2) can be written as (3), thanks to Lagrange multiplier.

(3) $$\underset{\alpha }{\arg\min}{\left\|x-D\alpha \right\|}_{2}^{2}+\lambda {\left\|\alpha \right\|}_{1}$$

where λ is a regularization parameter for tradeoff between reconstruction fidelity and sparsity. Equation (3) can be efficiently solved by basis pursuit and greedy pursuit algorithms, e.g., orthogonal matching pursuit (OMP).

### 2.3. GRSC

Motivated by the recent progress in sparse coding and manifold learning, GRSC algorithm is an efficient signal processing technique which explicitly considers the local geometrical structure of the data. To encode the geometrical information in the data, the GRSC algorithm builds a k−nearest neighbor graph to encode the geometrical information in the data. Hence, the graph Laplacian from the spectral graph theory can be used as a smooth operator to preserve the local manifold structure, which is incorporated into the sparse coding objective function as a regularizer. 

Let $X=\left[x_{1},x_{2},\ldots ,x_{m}\right]\in {ℜ}^{n\times m}$ be a data matrix containing m image patch vectors. The objective function of traditional sparse coding can be formulated as follows:

(4) $$\underset{A}{\arg\min}{\left\|X-DA\right\|}_{F}^{2}+\gamma \sum_{i=1}^{m}{\left\|\alpha_{i}\right\|}_{1}$$

where ${\left\|\cdot \right\|}_{F}$ denotes the Frobenius norm matrix, and A is the sparse coefficient matrix. The GRSC algorithm is based on the manifold assumption that if two data points $x_{i}$ and $x_{j}$ are close in the intrinsic geometry of the data distribution, the representation of this two points $\alpha_{i}$ and $\alpha_{j}$ with respect to the dictionary D should be also close to each other. For a set of given data points $x_{1},x_{2},\ldots ,x_{m}$, we can construct a nearest neighbor graph G with m vertices that represent the data points. Supposed that W is the weight matrix of the graph G. If the data point $x_{i}$ is among the k nearest neighbors of the data point $x_{i}$ or the data point $x_{j}$ is among the k nearest neighbors of the data point $x_{i}$, we define $W_{ij}=1$, otherwise, we define $W_{ij}=0$. Based on this, the degree of $x_{i}$ can be defined as $h_{i}=\sum_{j=1}^{m}W_{ij}$, and $H=diag\left(h_{1},\ldots ,h_{m}\right)$. Considering the problem of mapping the graph G to the sparse representation A, a good map can be obtained by minimize the following objective function:

(5) $$\frac{1}{2}\sum_{i=1}^{m}\sum_{j=1}^{m}{\left(\alpha_{i}-\alpha_{j}\right)}^{2}W_{ij}=Tr\left(ALA^{T}\right)$$

where L=H-W denotes the Laplacian matrix. Hence, the following objective function of the GRSC algorithm can be obtained by incorporating the Laplacian regularizer (5) into (4):

(6) $$\underset{A}{\arg\min}{\left\|X-DA\right\|}_{F}^{2}+\beta Tr\left(ALA^{T}\right)+\gamma \sum_{i=1}^{m}{\left\|\alpha_{i}\right\|}_{1}$$

where β is the regularization parameter. The optimization problem (6) can be solved following the method proposed in article [57].

## 3. Multitask Pansharpening Method: GRSC-ACD

**Figure 2:**
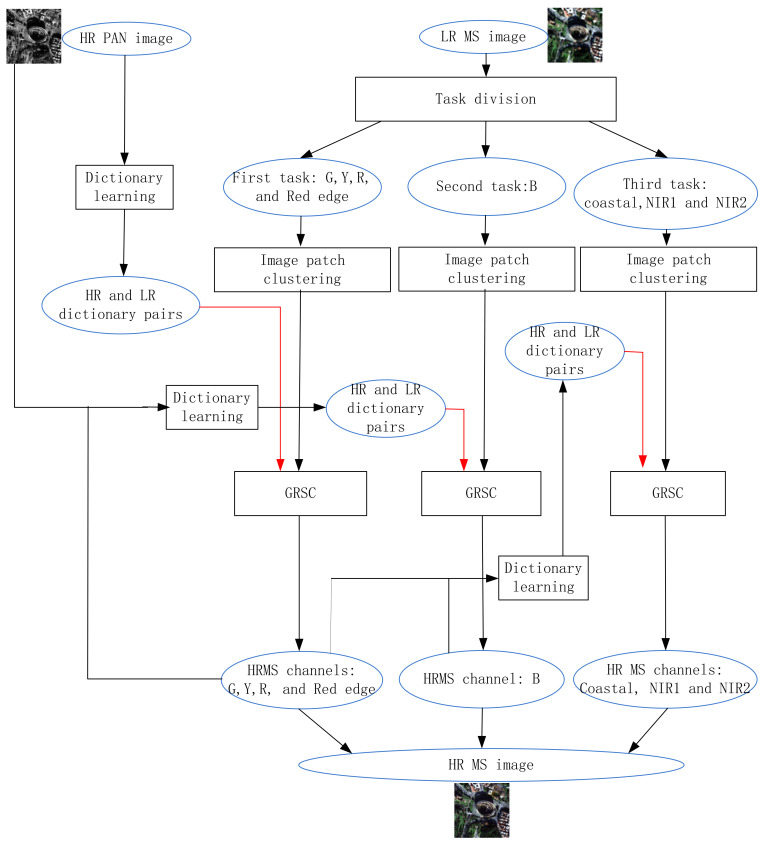
Scheme of proposed graph regularized sparse coding and adaptive coupled dictionary (GRSC-ACD) method.

In this section, we introduce the proposed multitask PS method GRSC-ACD for the WorldView-2 data. Figure 2 shows the scheme of the proposed method. The detailed algorithm steps of the proposed method are described as follows.

### 3.1. Description of Multitask Pansharpening

**Figure 3:**
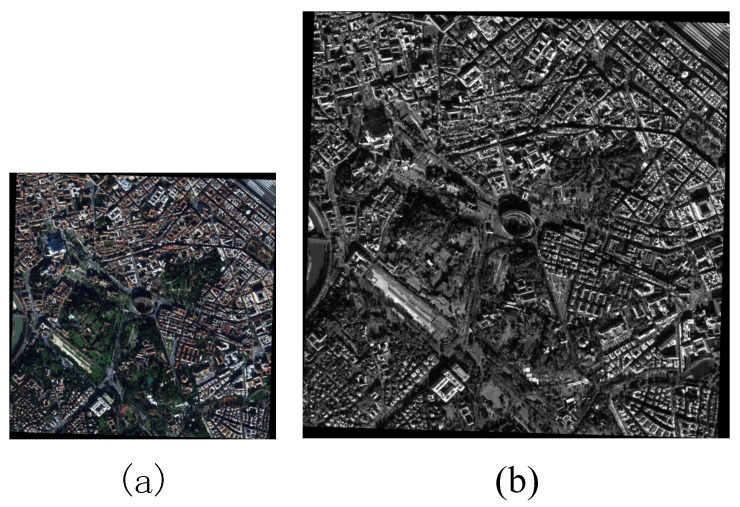
WorldView-2 data set. (**a**) MS image (1150×1151); (**b**) PAN image (4600×4604 ).

The first step of the proposed method is to divide the PS process into three tasks according to the degree of correlation among the MS channels and the PAN channel and the relative spectral response curves of different channels. The WorldView-2 data used in this paper is exhibited in Figure 3. Figure 3a shows the MS image with eight spectral bands with the size of 1150×1151, and Figure 3b shows the PAN image with the size of 4600×4604. Then, the degraded PAN image is obtained by blurring and downsampling the PAN image, which has the same spatial resolution and scale as the original MS image. The correlation coefficient matrix among the MS channels and the PAN channel is computed, which is listed in Table 1. According to the correlation coefficients of different channels and the relative spectral response curves among different channels as shown in Figure 1, the PS process of WorldView-2 data is divided into three tasks.

(1) First task: The correlation coefficients between the MS channels including green, yellow, red and red edge, and the PAN channel are listed in Table 1, which are highlighted in red. The green, yellow, red and red edge bands have high correlation to the PAN image; also, these bands are almost within the wavelength range covered by the PAN image. Hence, in the first task, these MS channels will be sharpened together. For this task, the HR PAN image and its degraded version are used to learn the coupled dictionary pair.
(2) Second task: In Figure 1, the blue band is mostly within the wavelength range covered by the PAN image. However, it has low correlation to the PAN image. Hence, the second task specially deals with the blue band. From the correlation coefficient labeled with blue color, the blue band and the green band have high correlation. Hence, the PAN image and the reconstructed green band are used as the dataset to learn the adaptive coupled dictionary for this task.
(3) Third task: The remaining MS channels, i.e., coastal, NIR1, and NIR2, are almost outside the wavelength range covered by the PAN image shown in Figure 1. In this task, three MS channels are divided into two groups: (1) coastal band; (2) NIR1 and NIR2. For these two groups, different reconstructed HR MS bands are chosen to learn the adaptive coupled dictionaries. From the correlation coefficient labeled with purple color, it can be concluded that the coastal band is highly related to the blue band. Hence, the reconstructed blue band is used to learn the coupled dictionary for sharpening the coastal band. The correlation coefficients labeled with green color show the high degree of correlation among red edge, NIR1, and NIR2. Hence, for sharpening the NIR1 and NIR2 bands, we use the reconstructed red edge band to train the coupled dictionary.

### 3.2. Pansharpening Algorithm via GRSC for Each Task

The purpose of PS is to generate an HR MS image $X^{H}$ with a LR MS image $X^{L}$ and an HR PAN image $P^{H}$. For each task, the MS channels have high correlation to each other. Hence, the image patches from these MS channels have the same or similar manifold structures. Let $X_{p,t}^{L}$ be the pth band of the LR MS bands for the tth task, where p=1,…,P, and t=1,…,T. Then, all the LR MS bands are tiled into image patches with the patch size of r×r and the overlapping size of s×s. Each image patch is arranged in a column vector, and all the column vectors form an image patch set that is denoted as $\Omega =\left[x_{1,1}^{L},\ldots ,x_{1,J}^{L},\ldots ,x_{p,1}^{L},\ldots ,x_{p,J}^{L},\ldots ,x_{P,1}^{L},\ldots ,x_{P,J}^{L}\right]\in {ℜ}^{r^{2}\times JP}$, where $x_{p,j}^{L}$ is the jth image patch of the pth spectral band of LR MS image. The PS process consists of three main steps which are described as follows.

(1) ***Constructing image patch sets with similar geometrical structure***: To acquire the precise sparse representations of the image patches, the set Ω is first separated into several subsets with K-means clustering algorithm. Let $\Omega_{b}$ be the subset of each class, where b=1,2,…,B, and B is the total number of the subsets. All the image patches in a subset share the same or similar local geometrical structures.
(2) ***Sparse coding of the subsets* via *GRSC***: The proposed method is based on the assumption that the LR MS image patch and its corresponding HR MS image patch share the same sparse representation over the coupled dictionary pair. Let $D^{L}$ and $D^{H}$ be the LR dictionary and the HR dictionary, respectively. The dictionary construction method will be introduced in the following subsection. Considering the graph regularized sparse coding for image representation, we first construct the weighted graph matrix $W_{b}$ and the degree matrix $H_{b}$ for the subset $\Omega_{b}$. Then, the Laplacian matrix can be defined as $L_{b}=H_{b}-W_{b}$. The sparse representation of the subset $\Omega_{b}$ can be estimated by solving the following objective function:
  (7) $$\underset{A_{b}}{\arg\min}{\left\|\Omega_{b}-D_{b}^{L}A_{b}\right\|}_{F}^{2}+\beta Tr\left(A_{b}L_{b}A_{b}{}^{T}\right)+\gamma \sum_{v=1}^{V_{b}}{\left\|\alpha_{b,v}\right\|}_{1}$$
  where $A_{b}$ is the sparse coefficient matrix for the subset $\Omega_{b}$, and $\alpha_{b,v}$ is the sparse vector of the vth image patch in the subset. β and γ are the regularization parameters to balance the contribution of the two regularization terms. To solve the objective function by optimizing over each $\alpha_{b,v}$, (7) can be rewritten in a vector form. First, the reconstruction error ${\left\|\Omega_{b}-D_{b}^{L}A_{b}\right\|}_{F}^{2}$ can be written as $\sum_{v=1}^{V_{b}}{\left\|x_{b,v}-D_{b}^{L}\alpha_{b,v}\right\|}_{2}^{2}$. The Laplacian regularizer $Tr\left(A_{b}L_{b}A_{b}{}^{T}\right)$ can be rewritten as follows:
  (8) $$\begin{array}{l} \mathrm{Tr}\left(A_{b}L_{b}A_{b}{}^{T}\right)=\mathrm{Tr}\left(\sum_{v,u=1}^{V_{b}}L_{v,u}\alpha_{b.v}\alpha_{b,u}^{T}\right)\\ \;\;\;\;\;=\sum_{v,u=1}^{V_{b}}L_{v,u}\alpha_{b,u}^{T}\alpha_{b,v}^{}=\sum_{v,u=1}^{V_{b}}L_{v,u}\alpha_{b,v}^{T}\alpha_{b,u}^{} \end{array}$$
  Then, by combining (7) and (8), the problem (5) can be written as
  (9) $$\min\sum_{v=1}^{V_{b}}{\left\|x_{b,v}-D_{b}^{L}\alpha_{b,v}\right\|}_{2}^{2}+\beta \sum_{v,u=1}^{V_{b}}L_{v,u}\alpha_{b,v}^{T}\alpha_{b,u}^{}+\gamma {\left\|\alpha_{b,v}\right\|}_{1}$$
  Based on the feature-sign search algorithm proposed in [59], the problem in (9) can be effectively solved to acquire the optimal sparse coefficient matrix $A_{b}$.
(3) ***Reconstructing the HR MS channels for each task***: The estimated sparse coefficient matrix ${\hat{A}}_{b}$ for the subset $\Omega_{b}$ can be obtained by solving the problem in (9). Then, the HR MS image patch subset $\Omega_{b}^{H}$ corresponding to $\Omega_{b}$ can be calculated through the following Formula (10).
  (10) $$\Omega_{b}^{H}=D_{b}^{H}{\hat{A}}_{b}$$

After all the HR MS image patch subsets are obtained, the MS bands for each task can be reconstructed by averaging the overlapped image patches.

### 3.3. Dictionary Learning

Dictionary learning is an essential step in the proposed GRSC-ACD method. For dif-ferent tasks, different coupled dictionary pairs need to be learned according to the charac-teristic of the MS channels. In our method, the images used to learn the coupled dictionary should be updated according to the characteristics of the tasks. The detailed descriptions are as follows.

(1) First task: This task processes the MS channels: green, red, yellow, and red edge. These MS bands are within the wavelength range covered by the PAN image and show high correlation to the PAN image. Hence, the HR PAN image and its degraded version are suitable to learn the coupled dictionary pair for the first task.
(2) Second task: This task only processes the blue band, which is partially outside the wavelength band covered by the PAN image, and has low correlation to the PAN image. Thus, only using the PAN image to learn the coupled dictionary is not suitable for this task. To effectively represent the image patches subsets, the PAN image and the reconstructed HR green band with high correlation to the blue band are selected to learn the coupled dictionary.
(3) Third task: This task sharpens the MS channels that are almost outside the wavelength range covered by the PAN image, i.e., coastal, NIR1, and NIR2. As shown in Table 1, the coastal band has very low correlation to the NIR1 and NIR2 bands. Hence, this task is divided into two subtasks. One subtask processes the coastal spectral band. For this subtask, the reconstructed blue band is used to learn the coupled dictionary. Another subtask processes the NIR1 and NIR2 bands. For this subtask, the reconstructed red edge band is used to learn the coupled dictionary.

Then, the dictionary construction method for each subset $\Omega_{b}$ is introduced. Let $Y_{k,b}^{H}$, k=1,2,…,K be high-resolution images for dictionary learning. The HR images are blurred and downsampled to obtain the corresponding LR images $Y_{k,b}^{L}$, k=1,2,…,K. Then, the HR and LR image pairs are tiled into the image patches. The patch size for the LR images is r×r, and the overlapping size is s×s. While the patch size for the HR images is $F_{DS}r\times F_{DS}r$, and the overlapping size is $F_{DS}s\times F_{DS}s$, where $F_{DS}$ is the scale factor between MS and PAN. The image patches are arranged into vectors; hence, the coupled dictionary is constructed by the raw LR and HR image patch vectors, which are defined as $D_{b}^{L}$ and $D_{b}^{H}$, respectively.

In short, our algorithm can be summarized in Algorithm 1.
**Algorithm 1.** The GRSC-ACD Pansharpening Method.**Input:** LR MS image $X^{L}$, PAN image $P^{H}$**Initialization:** Set parameters β, γ, r, s and B1: Split the PS process into multiple tasks according to the relative spectral response as shown in Figure 1 and the channel correlation matrix as listed in Table 12: **for**
t←1,2,…,T
**do**3: Separate all the MS bands $X_{p,t}^{L}$,p=1,…,P into image patches and form an image patch set Ω4: Generate each subset $\Omega_{b}$, b=1,2,…,B using K-means clustering algorithm5: **for** b←1,2,…,B
**do**6:  Learn the LR dictionary $D_{b}^{L}$ and the HR dictionary $D_{b}^{H}$7:  Compute the sparse coefficient matrix ${\hat{A}}_{b}$ according to (7)8:  Compute the HR image patch subset $\Omega_{b}^{H}$ through (10)9: **end for**10: Generate the HR MS bands $X_{p,t}^{H}$, p=1,…,P11: **end for****Output:** Target HR MS image $X^{H}$.

## 4. Experiments

### 4.1. Experimental Dataset and Comparison Methods

Figure 3 shows a pair of images from WorldView-2, i.e., an eight-band MS image and a one-band PAN image. These two images were acquired over Rome on December 10, 2009. The original MS image contains 1150×1151 pixels with a spatial resolution of 2 m, and the HR PAN image contains 4600×4604 pixels with a spatial resolution of 0.5 m. In the following experiments, the degraded and real images are used to evaluate the performance of the proposed method. First, we crop an MS image containing 256×256 pixels and a PAN image containing 1024×1024 pixels. For the degraded experiments, an HR reference image is required for quality assessment. To achieve this goal, the original MS and PAN images are blurred and downsampled to the corresponding LR versions with resolutions of 8 m and 2 m, respectively. The original MS image is regarded as the reference image. For the real experiments, we crop a MS image with a resolution of 2 m containing 100×100 pixels and a PAN image with a resolution of 0.5 m containing 400×400 pixels.

To verify the fusion performance of the proposed method, ten PS methods are taken for performance comparison. These methods include the GS algorithm [5], the high-pass filter (HPF) algorithm [60], the partial replacement adaptive component substitution (PRACS) algorithm [8], the MTF-GLP with high-pass modulation (MTF-GLP-HPM) algorithm [18], the band-dependent spatial-detail (BDSD) algorithm [61], the proportional additive wavelet to the luminance component with haze correction (AWLPH) algorithm [62], the robust BDSD (RBDSD) algorithm [63], the PN-TSSC algorithm [40], the OCDL algorithm [41], and the GRSC algorithm [56]. The key parameters of these methods refer to the corresponding articles. In addition, a resampled MS image is also included during the comparison and is referred as EXP.

### 4.2. Quality Assessment Indexes

To quantitatively evaluate the fusion performance, various quality indexes are used. Six quality indexes are used in the simulated experiments, including root-mean-square-error (RMSE), average spectral mapper (SAM) [64], erreur relative globale adimensionnelle de synthese (ERGAS) [65], Q [66], structural similarity index (SSIM) [67], and Q2n [68] are considered. The ideal values of RMSE, SAM, ERGAS, Q, SSIM, and Q2n are 0, 0, 0, 1, 1, and 1, respectively. The “quality with no reference” (QNR) [69] is used in the real experiments to assess the fusion performance. The QNR index consists of the spectral distortion index $D_{\lambda }$ and the spatial distortion index $D_{s}$. The best values of $D_{\lambda }$ and $D_{s}$ are both 0, while the best value of QNR is 1.

### 4.3. The Choice of Tunning Parameters

For our method, its performance is affected by several tunning parameters, i.e., regularization parameters β and γ, patch size, and overlapping size. To optimize the parameters for better performance, experiments with different parameters are conducted on the degraded and real data, respectively.

#### 4.3.1. Regularization Parameters

**Figure 4:**
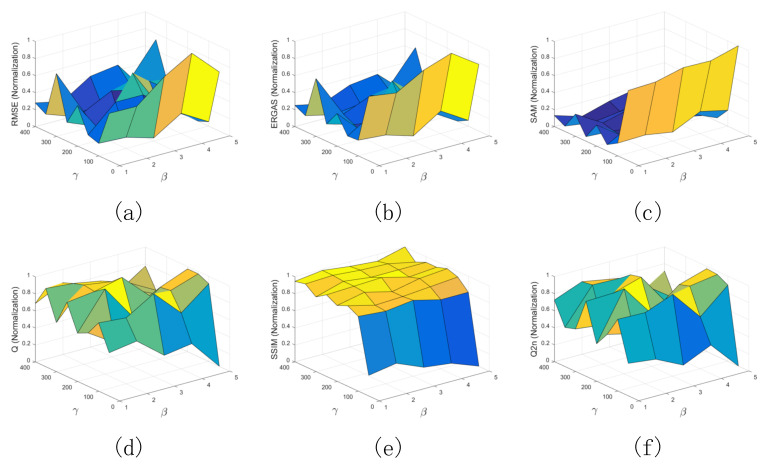
Performance analysis of proposed method with different regularization parameters on degraded data. (**a**) RMSE; (**b**) ERGAS; (**c**) SAM; (**d**) Q; (**e**) SSIM; (**f**) Q2^n^.

In this section, the effects of regularization parameters β and γ on the fusion performance are explored. For the degraded data, the patch size is first set to 7×7, and the overlapping size is set to 3. For the regularization parameter β from 1–5 at an interval of 1, and the regularization parameter γ from 50–400 at an interval of 50, their influence on the performance of the proposed method is studied. Six quality indexes are calculated, where the average RMSE of eight bands is presented. In addition, all the values of the quality indexes are normalized to the range of [0, 1]. The normalized results with respect to different parameters are plotted in Figure 4, where the X axis, Y axis, and Z axis stand for the regularization parameter β, the regularization parameter γ, and the normalized results, respectively. The smaller the RMSE, ERGAS, and SAM values, the better the fused results. The larger the Q, SSIM, and Q2n values, the better the fused results. In Figure 4, the proposed method achieves better performance for the degraded data when the regularization parameter β is set to 3 and the regularization parameter γ is set to 250.

**Figure 5:**
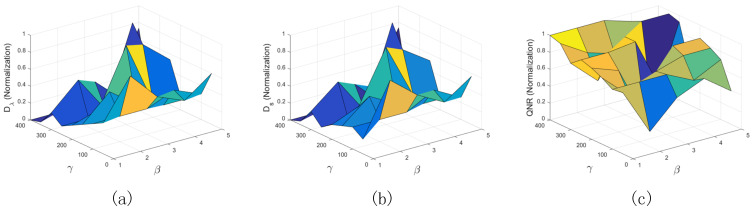
Performance analysis of proposed method with different regularization parameters on real data. (**a**) $D_{\lambda }$; (**b**) $D_{s}$; (**c**) QNR.

For the real data, the influence of the regularization parameters on the performance of the proposed method is also discussed. In the experiment, the real MS image has the size of 100×100 and the real PAN image has the size of 400×400. The patch size is set to 25×25, and the overlapping size is set to 1/4 of patch size. For the regularization parameter β from 1–5 at an interval of 1, and the regularization parameter γ from 50–400 at an interval of 50, their influence on the performance of the proposed method is studied. Three indexes including $D_{\lambda }$, $D_{s}$, and QNR are used to evaluate the quality of the pansharpened results. All the quality indexes are normalized to the range of [0, 1]. The normalized results with respect to different parameters are plotted in Figure 5, where the X axis, Y axis, and Z axis stand for the regularization parameter β, the regularization parameter γ, and the normalized results, respectively. The smaller the values of $D_{s}$ and $D_{\lambda }$, the better the fused image. The larger the value of QNR, the better the fused image. Figure 5 shows that the proposed method achieves better performance for the real data when the regularization parameter β is set to 3 and the regularization parameter γ is set to 250.

#### 4.3.2. Patch Size and Overlapping Size 

**Figure 6:**
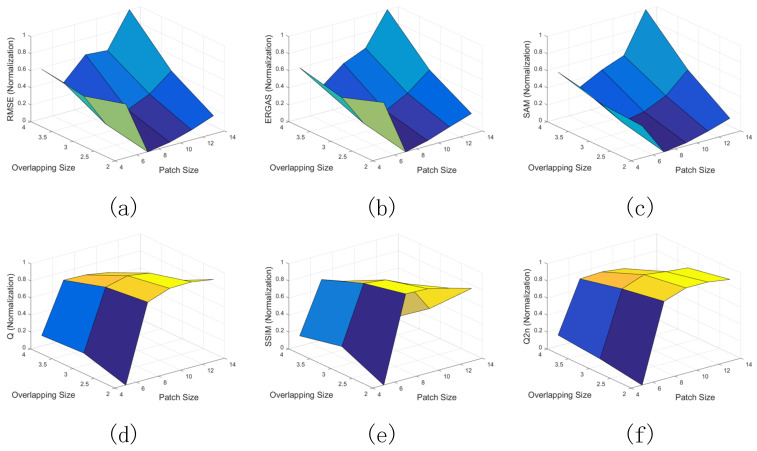
Performance analysis of proposed method under different patch sizes and overlapping sizes on degraded data. (**a**) RMSE; (**b**) ERGAS; (**c**) SAM; (**d**) Q; (**e**) SSIM; (**f**) Q2^n^.

In this section, the effects of the patch size and the overlapping size are investigated. For the degraded data, the regularization parameter β is set to 3, and the regularization parameter γ is set to 250. Five patch sizes for the LR MS image, including 5×5, 7×7, 9×9, 11×11, and 13×13, and three overlapping sizes, including 2, 3, 4, are considered together. The performance surface of the proposed method under different patch sizes and overlapping sizes is exhibited in Figure 6, where the X axis, Y axis, and Z axis indicate the patch sizes, the overlapping sizes, and the normalized results, respectively. In Figure 6, the proposed method provides the optimal RMSE, ERGSA, SAM, Q, SSIM, and Q2n values, when the patch size is set to 7×7 and the overlapping size is set to 2. However, our proposed method with smaller overlapping size needs more computational time. Hence, considering the tradeoff between the pansharpening quality and running time, the patch size and overlapping size are respectively set to 7×7 and 3 in the following experiments.

**Figure 7:**
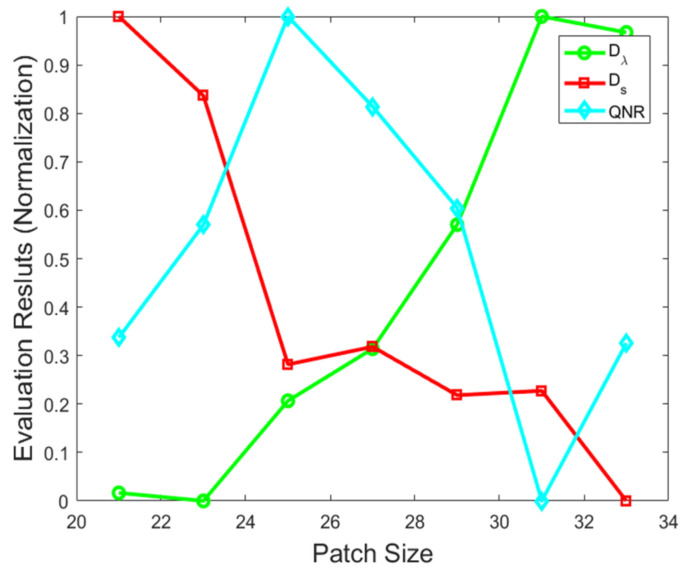
Performance analysis of proposed method under different patch sizes on real data.

For the real data, the effect of the patch size on the performance of the proposed method is discussed and analyzed. In the experiment, the regularization parameter β is set to 3, and the regularization parameter γ is set to 250. For the patch size varying from 21–33 at an interval of 2, the quality curves of the proposed method under different patch size are plotted in Figure 7. The green, blue, and red lines represent three quality indexes, i.e., $D_{s}$, $D_{\lambda }$, and QNR, respectively. The proposed method obtains the best QNR value when the patch size is 25×25; hence, in the following experiments, the patch size for real data is set to 25×25.

### 4.4. Experimental Results on Degraded Images

**Figure 8:**
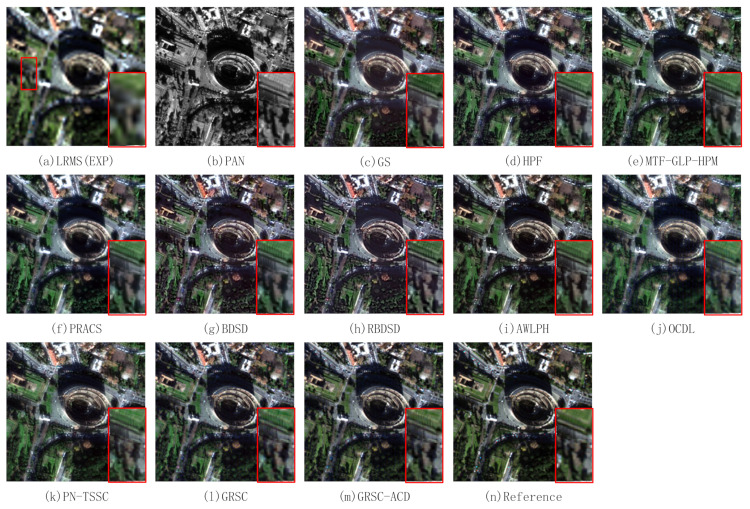
Pansharpened results of different PS methods on first pair of degraded images.

In this section, the proposed method is evaluated on two pairs of degraded WorldView-2 images. The input images and the pansharpened results of different PS methods are shown in Figure 8. A local region is magnified and put at the bottom-right of each figure. Figure 8a,b exhibit the LR MS image with a resolution of 8 m and the PAN image with a resolution of 2 m. The LR MS image is obtained by the EXP method. The fused image of EXP has poor spatial resolution and good spectral resolution. Figure 8c–m illustrates the fused images of the GS, HPF, MTF-GLP-HPM, PRACS, BDSD, RBDSD, AWLPH, OCDL, PN-TSSC, GRSC, and GRSC-ACD methods, respectively. Figure 8n is the reference image. In terms of visual analysis, the fused images of the GS, HPF, MTF-GLP-HPM, PRACS, BDSD, RBDSD, AWLPH, OCDL, PN-TSSC, and GRSC methods suffer from slight spectral distortion, especially in vegetation areas. From the magnified region, the fused images of the OCDL and PN-TSSC methods, as shown in Figure 8j,k, exhibit slight blurring effects. The fused images of the BDSD and RBDSD methods, as shown in Figure 8g,h, show an oversharpening effect in spatial detail preservation. Figure 8m shows the fused image of the proposed GRSC-ACD method. Compared with the reference image and the fused images of the other methods, the proposed GRSC-ACD method achieves better spatial and spectral qualities in the fused image.

Table 2 lists the quantitative evaluation results of the fused images of different methods shown in Figure 8, where the best value of each index is highlighted in bold and the second best value of each index is underlined. Table 2 shows that the proposed GRSC-ACD method obtains the best RMSE, ERGAS, Q, SSIM, and Q2n values. However, the proposed method is inferior to the MTF-GLP-HPM method in terms of SAM.

**Figure 9:**
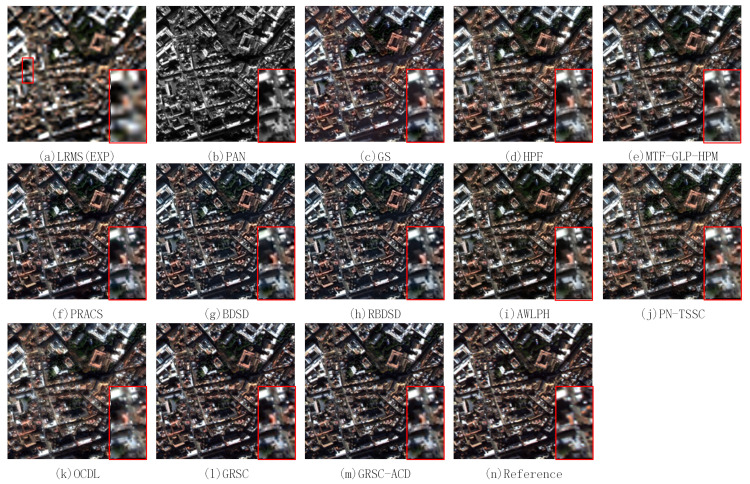
Pansharpened results of different PS methods on second pair of degraded images.

Figure 9 illustrates the pansharpened results of the second pair of degraded images. A magnified region is put at the bottom-right of each figure. Figure 9a,b show the resampled MS image obtained by the EXP method and the PAN image, respectively. The fused image of the EXP method has poor spatial quality. The reference image is shown in Figure 9n. Figure 9c shows the fused image of the GS method, which exhibits a slight spectral distortion as compared with the reference image. Figure 9d–l illustrates the fused images of the HPF, MTF-GLP-HPM, PRACS, BDSD, RBDSD, AWLPH, OCDL, PN-TSSC, and GRSC methods. The fused images of these methods are comparable to the reference image in preserving the spectral information. From the magnified region, the HPF, MTF-GLP-HPM, PRACS, BDSD, RBDSD, AWLPH, and GRSC methods are capable of effectively preserving the spatial details. Compared with the reference image, the pansharpened images produced by the PN-TSSC and OCDL methods suffer from a slight spatial detail distortion. The fused image of the proposed GRSC-ACD method is shown in Figure 9m, which shows good spectral and spatial qualities.

Table 3 lists the quantitative evaluation results of the pansharpened images shown in Figure 9, where the best values are labeled in bold, and the second best values are underlined. The proposed GRSC-ACD method obtains the best values in terms of the RMSE, SAM, Q, SSIM, and Q2n indexes. Regarding the ERGAS index, the PN-TSSC method obtains the best value, and the proposed method obtains the second best value. In general, the proposed method achieves better fusion performance for the degraded datasets based on the subjective and objective assessments.

### 4.5. Analysis of Difference Images

**Figure 10:**
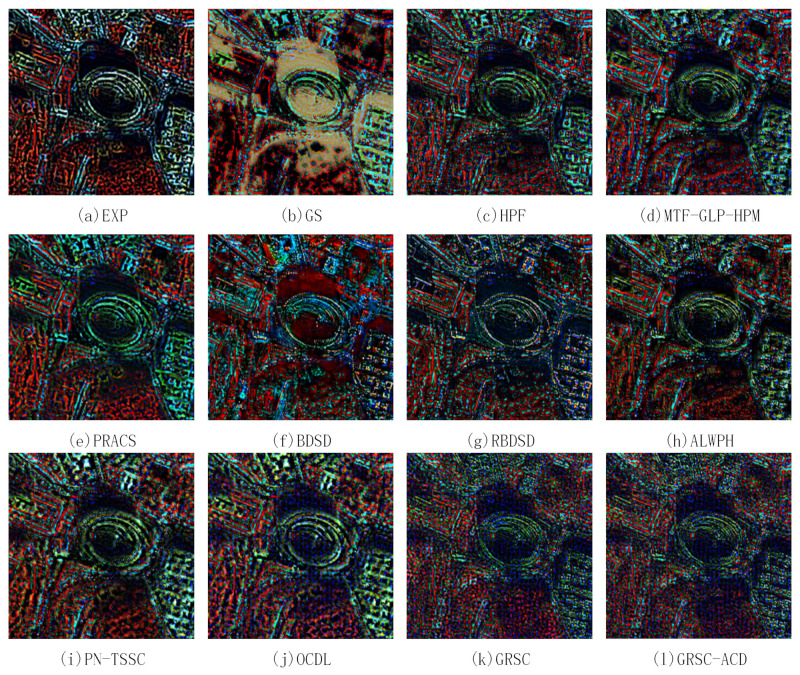
False color difference images of pansharpened results shown in Figure 8 (Selected bands: 7 (NIR1), 4 (yellow), and 1 (Coastal)).

**Figure 11:**
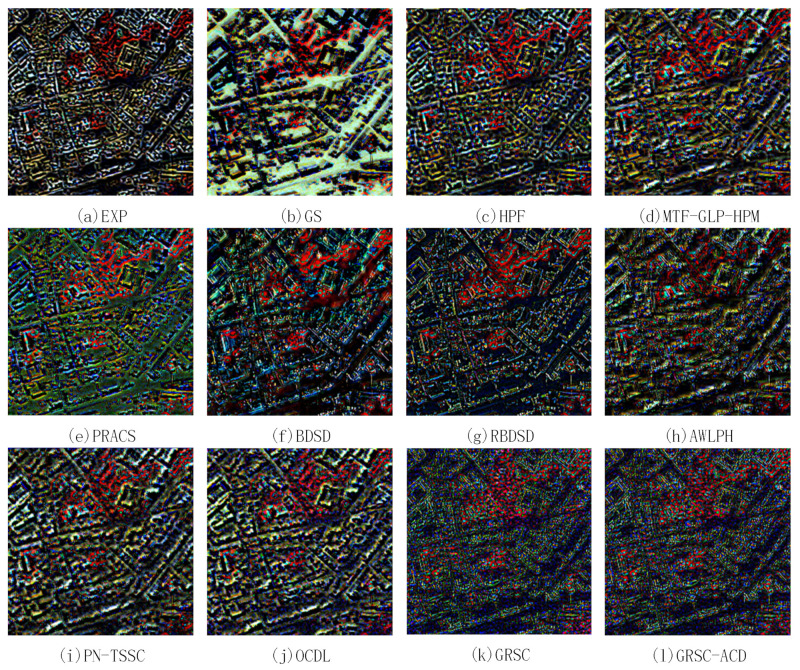
False color difference images of pansharpened results shown in Figure 9 (Selected bands: 7 (NIR1), 4 (yellow), and 1 (Coastal)).

The above section only gives the global assessments of the fused results. To better understand where the reconstruction errors are localized, the difference images between the pansharpened images and the reference image for two pairs of degraded images are calculated and analyzed. Figure 10 and Figure 11 show the false color difference images of the fused images shown in Figure 8 and Figure 9, respectively. The RGB channels are composed of 7 (NIR1), 4 (yellow), and 1 (coastal). In Figure 10 and Figure 11, black color means zero difference, while intense red, blue, and green colors mean obvious errors in NIR1, yellow, and coastal channels, respectively. In [43], the abrupt color jumps between black colors are regarded as resolution loss. From this point of view, if the abrupt changes have wider transition region, the resolution loss will be more severe.

In Figure 10 and Figure 11, all the difference images have clear structures, indicating that all the methods produce a certain amount of resolution loss. Figure 10a,b and Figure 11a,b exhibit the difference maps of the EXP and GS methods, which have the widest transition regions as compared with the other difference maps. Thus, the EXP and GS methods produce more resolution loss than the other methods. Figure 10c–j and Figure 11c–j show the difference images of the HPF, MTF-GLP-HPM, PRACS, BDSD, RBDSD, ALWPH, PN-TSSC, and OCDL methods. The transition regions for the RBDSD and AWLPH methods are narrower than those for the HPF, MTF-GLP-HPM, PRACS, PN-TSSC, and OCDL methods. Therefore, the RBDSD and AWLPH can preserve the image resolution better. The difference images of the GRSC and GRSC-ACD methods are shown in Figure 10k–l and Figure 11k–l, respectively. The transition regions for the GRSC-ACD and GRSC methods are the narrowest; hence, the GRSC-ACD and the GRSC methods can preserve better image resolution as compared with the other methods.

In terms of spectral distortion, the EXP and GS methods performs the worst, because obvious dominating color appears. For the other PS methods, the intense red, blue, and green colors mainly occur at the boundaries of the objects. This indicates that the boundaries of the objects have severe spectral distortion, which may be associated with the resolution loss. In general, the proposed GRSC-ACD method and the GRSC method outperform the other methods in terms of preserving the spectral information.

### 4.6. Experimental Results of Real Images

**Figure 12:**
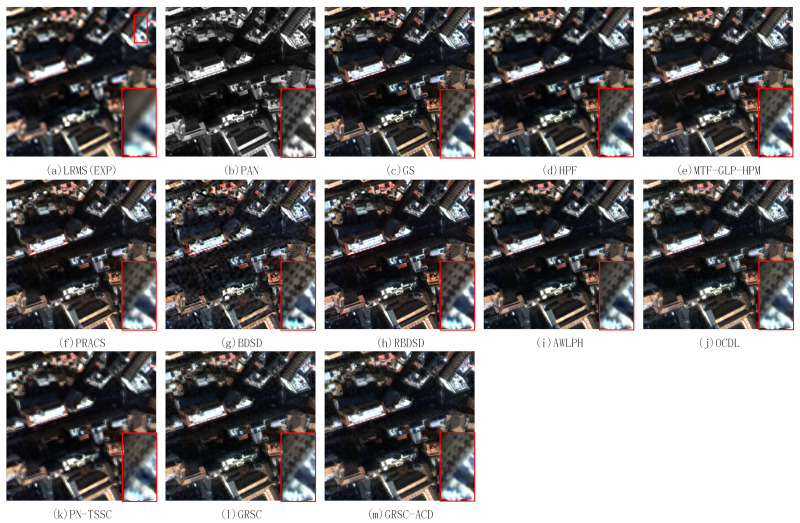
Pansharpened results of different PS methods on first pair of real images.

**Figure 13:**
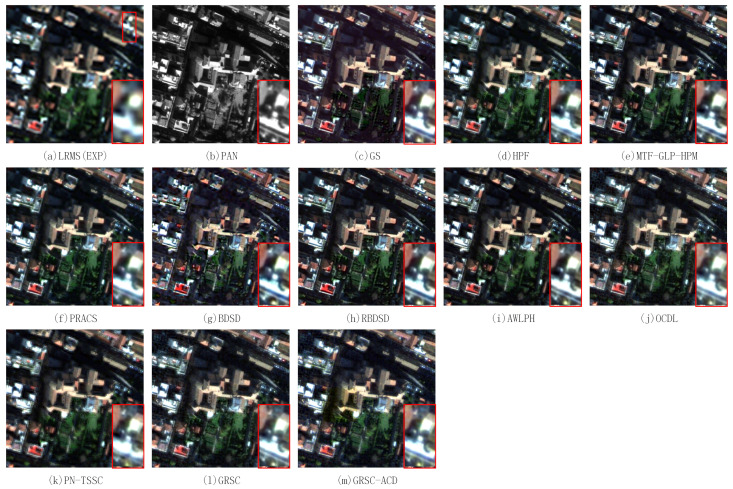
Pansharpened results of different PS methods on second pair of real images.

**Figure 14:**
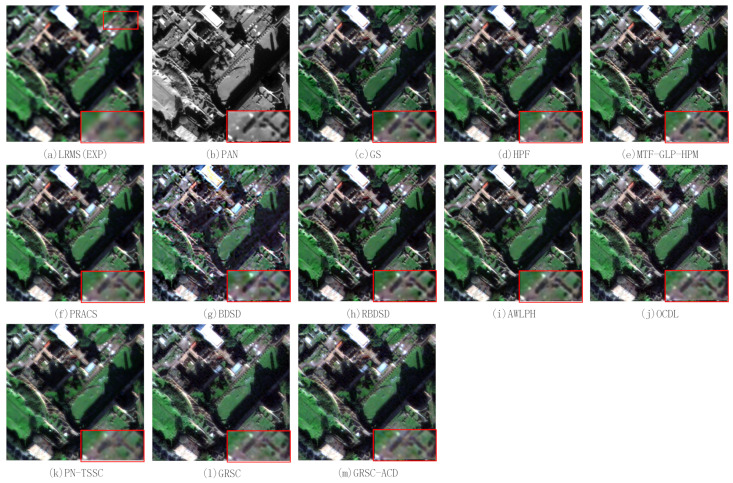
Pansharpened results of different PS methods on third pair of real images.

To further demonstrate the effectiveness of the proposed method, the proposed method is performed on three pairs of real images. The first pair of real images contains buildings. The second pair of real images contains buildings and vegetations. The third pair of real images contains buildings and vegetations. The fused images of different methods are shown in Figure 12, Figure 13 and Figure 14, respectively. For better visual comparison, we extract the local magnified region from each figure and put them at the right bottom of each figure. Figure 12a,b shows the resampled MS image and the PAN image, respectively. The pansharpened images of different PS methods on the first pair of real images are shown in Figure 12c–m. The fused image of EXP still has poor spatial resolution and good spectral quality. All the pansharpened methods show good ability to preserve the spectral information as compared with that of the fused image of EXP. From the magnified region, the fused images of the GS, MTF-GLP-HPM, PRACS, RBDSD, AWLPH, OCDL, GRSC, and GRSC-ACD exhibit good spatial qualities, and the fused images of the HPF, BDSD, and PN-TSSC suffer from blurring effects and spatial distortions.

Figure 13 shows the pansharpened images of different PS methods on the second pair of real images. Figure 13a is the fused image of EXP, which shows poor spatial resolution. The fused image of the GS method, as shown in Figure 13c, suffers from spectral distortion in the vegetation area. The fused images of the other PS methods, as shown in Figure 13d–m, exhibit natural colors as compared with the resampled MS image (EXP). From the magnified region, the fused images of the HPF and PN-TSSC methods exhibit slight blurring effects and artifacts. The fused images of the GS, MTF-GLP-HPM, PRACS, BDSD, RBDSD, AWLPH, OCDL, GRSC, and GRSC-ACD methods have good spatial qualities.

Table 4 lists the associated quantitative results of different methods on the first and second pairs of real datasets, where the best values are labeled in bold, and the second best values are underlined. For the first pair of real data, the GRSC-ACD method provides the second best $D_{\lambda }$ value and the best QNR value. The AWLPH method obtains the best $D_{s}$ value. The OCDL method obtains the second best values in terms of $D_{s}$ and QNR. For the second pair of real data, the GS method obtains the second best $D_{\lambda }$ value. Besides, the AWLPH method obtains the best $D_{s}$ value, and the proposed GRSC-ACD method obtains the best QNR value. The GRSC method obtains the second best value in terms of $D_{s}$, and the OCDL method obtains the second best value in terms of QNR.

The pansharpened results of different methods on the third pair of real images are shown in Figure 14. Figure 14a shows the resampled MS image, which has poor spatial resolution and good spectral quality. Figure 14c–m shows the pansharpened results of all the methods. Compared with the fused image of EXP, the fused images of the GS, HPF, MTF-GLP-HPM, PRACS, RBDSD, AWLPH, OCDL, PN-TSSC, GRSC, and GRSC-ACD methods exhibit good spectral preservation. However, the fused image of BDSD shows poor spectral quality. From the magnified region, the fused images of BDSD, OCDL, and PN-TSSC possess slight blurring artifacts. The fused images of GS, HPF, MTF-GLP-HPM, PRACS, RBDSD, AWLPH, and GRSC have comparable spatial quality as compared with that of the PAN image. The fused image of the proposed method, as shown in Figure 14m, gives impressive spectral and spatial qualities. Table 5 lists the associated quantitative results of Figure 14, where the best values are labeled in bold, and the second values are underlined. The proposed method accomplishes the second best $D_{\lambda }$ and $D_{s}$ values and the optimal QNR value. In short, the proposed GRSC-ACD method has better fusion performance than the other methods on the real data.

### 4.7. Algorithm Exceution Time Analsysis

In this section, we compare the computational time of the proposed method with the other PS methods. Table 2, Table 3, Table 4 and Table 5 list the computational time of five datasets. All the algorithms are implemented in MATLAB R2016a on a personal computer with 32 GB-RAM, Intel Xeon W-2125 CPU @4.00 GHz. From Table 2, Table 3, Table 4 and Table 5, the EXP, GS, HPF, MTF-GLP-HPM, PRACS, BDSD, RBDSD, and AWLPH methods take computational times less than 1 s. The computational time of the OCDL, PN-TSSC, GRSC, and GRSC-ACD algorithms is higher than that of the above methods because these methods adopt the sparse representation techniques. Parallel processing strategy can be applied to overcome this problem. Although our method takes the highest computational time, it has superior performance for sharpening the Worldview-2 data.

## 5. Conclusions

A multitask pansharpening method for the Worldview-2 data via graph regularized sparse coding and adaptive coupled dictionaries is proposed in this paper. We fully consider the spectral and correlation characteristics of the MS and PAN images and separate the pansharpening process into three tasks. The first task processes the MS channels that are fully overlapped by the PAN band. The second task processes the blue channel that is partially outside the wavelength range covered by the PAN band. The third task processes the channels that are almost outside the wavelength range covered by the PAN band. For each subtask, the interband and intraband correlations among image patches are considered. For different subtasks, suitable coupled dictionary pairs are designed to efficiently represent the image patch subsets. A variety of experiments are conducted, and the experimental results demonstrate that the proposed method achieves better performance for sharpening the WorldView-2 data.

## Figures and Tables

**Table 1:** Channel mutual correlation coefficient matrix.

	PAN	Coastal	Blue	Green	Yellow	Red	Red Edge	NIR1	NIR2
PAN	1.0000	0.7493	0.8811	0.9629	0.9636	0.9535	0.9493	0.8253	0.8193
Coastal	0.7493	1.0000	0.9494	0.8317	0.7464	0.6974	0.6337	0.5072	0.4973
Blue	0.8811	0.9494	1.0000	0.9552	0.8897	08636	0.7584	0.6020	0.5887
Green	0.9629	0.8317	0.9552	1.0000	0.9675	0.9578	0.8679	0.7110	0.6982
Yellow	0.9636	0.7464	0.8897	0.9675	1.0000	0.9843	0.8825	0.6886	0.6855
Red	0.9535	0.6974	0.8636	0.9578	0.9843	1.0000	0.8620	0.6772	0.6682
Red Edge	0.9493	0.6337	0.7584	0.8679	0.8825	0.8620	1.0000	0.9357	0.9352
NIR1	0.8253	0.5072	0.6020	0.7110	0.6886	0.6772	0.9357	1.0000	0.9928
NIR2	0.8193	0.4973	0.5887	0.6982	0.6855	0.6682	0.9352	0.9928	1.0000

**Table 2:** Quantitative evaluation results of fused images of different methods shown in Figure 8.

Methods	RMSE	ERGAS	SAM	Q	SSIM	Q2n	Time(s)
EXP	48.7750	5.7132	5.0109	0.8222	0.8441	0.8145	0.004
GS	41.9282	5.0331	6.5103	0.8735	0.9207	0.8574	0.09
HPF	35.7340	4.2492	4.9089	0.9139	0.9306	0.9102	0.06
MTF-GLP-HPM	31.2260	3.6602	**4.0269**	0.9289	0.9475	0.9274	0.28
PRACS	36.2372	4.3248	5.0098	0.9102	0.9251	0.9108	0.22
BDSD	48.0075	4.6408	6.3423	0.8961	0.9216	0.8871	0.09
RBDSD	45.6704	5.4831	6.1062	0.8817	0.9053	0.8680	0.19
AWLPH	38.5274	4.5192	4.7565	0.9193	0.9331	0.9148	0.17
OCDL	36.4954	4.3003	4.8793	0.8767	0.9252	0.8648	67.16
PNTSSC	44.4959	4.2806	4.9471	0.9069	0.9278	0.9065	4.22
GRSC	30.5811	3.5347	4.9572	0.9293	0.9401	0.9223	142.53
GRSC-ACD	**28.5203**	**3.3265**	4.1763	**0.9353**	**0.9484**	**0.9305**	147.63

**Table 3:** Quantitative evaluation results of fused images of different methods shown in Figure 9.

Methods	RMSE	ERGAS	SAM	Q	SSIM	Q2n	Time (s)
EXP	66.1508	6.9654	5.2397	0.7948	0.7749	0.7931	0.004
GS	48.1403	5.1607	5.6282	0.8957	0.9245	0.8876	0.08
HPF	39.4451	4.2435	4.3694	0.9340	0.9336	0.9322	0.07
MTF-GLP-HPM	36.1436	3.8433	4.2311	0.9451	0.9463	0.9437	0.29
PRACS	34.8559	3.9944	4.6390	0.9429	0.9436	0.9474	0.23
BDSD	44.9257	4.6012	4.9426	0.9306	0.9301	0.9297	0.09
RBDSD	38.6037	4.0112	4.0298	0.9468	0.9421	0.9454	0.18
AWLPH	40.7509	4.3785	4.3772	0.9498	0.9432	0.9492	0.15
OCDL	44.6863	4.7780	4.7649	0.9006	0.9181	0.8987	54.14
PN-TSSC	44.4959	**2.2806**	4.9471	0.9069	0.9278	0.9065	4.98
GRSC	28.8187	3.0460	3.9993	0.9656	0.9587	0.9615	139.24
GRSC-ACD	**27.5646**	2.9205	**3.6299**	**0.9659**	**0.9625**	**0.9672**	151.27

**Table 4:** Quantitative evaluation results of fused images of different methods shown in Figure 12 and Figure 13.

Methods	Real Dataset 1				Real Dataset 2			
	$D_{\lambda }$	$D_{s}$	QNR	Time(s)	$D_{\lambda }$	$D_{s}$	QNR	Time (s)
EXP	**0.0000**	0.1403	0.8597	0.005	**0.0000**	0.1263	0.8737	0.005
GS	0.0148	0.0785	0.9078	0.23	0.0122	0.0722	0.9165	0.21
HPF	0.0312	0.0530	0.9175	0.21	0.0360	0.0505	0.9153	0.18
MTF-GLP-HPM	0.0184	0.0489	0.9335	0.43	0.0265	0.0495	0.9253	0.42
PRACS	0.0139	0.0539	0.9330	0.80	0.0133	0.0530	0.9344	0.74
BDSD	0.0145	0.0725	0.9141	0.13	0.0183	0.0472	0.9354	0.15
RBDSD	0.0081	0.0592	0.9332	0.49	0.0144	0.0597	0.9268	0.34
AWLPH	0.0279	**0.0388**	0.9344	0.27	0.0282	**0.0371**	0.9357	0.26
OCDL	0.0145	0.0426	0.9435	145.70	0.0195	0.0442	0.9371	163.12
PN-TSSC	0.0164	0.0619	0.9228	28.23	0.0153	0.0518	0.9337	26.08
GRSC	0.0133	0.0541	0.9332	222.08	0.0278	0.0435	0.9299	223.86
GRSC-ACD	0.0055	0.0501	**0.9447**	399.01	0.0128	0.0467	**0.9411**	353.69

**Table 5:** Quantitative evaluation results of fused images of different methods shown in Figure 14.

Methods	Real Dataset 3			
	$D_{\lambda }$	$D_{s}$	QNR	Time (s)
EXP	**0.0000**	0.1155	0.8845	0.002
GS	0.0318	0.0987	0.8727	0.28
HPF	0.0309	0.0623	0.9087	0.20
MTF-GLP-HPM	0.0403	0.0782	0.8847	0.56
PRACS	0.0204	0.0721	0.9090	0.81
BDSD	0.0288	**0.0453**	0.9272	0.14
RBDSD	0.0257	0.0950	0.8817	0.58
AWLPH	0.0427	0.0689	0.8914	0.30
OCDL	0.0295	0.0614	0.9109	159.05
PN-TSSC	0.0264	0.0505	0.9245	22.31
GRSC	0.0385	0.0514	0.9121	217.87
GRSC-ACD	0.0121	0.0481	**0.9403**	318.06

## Data Availability

Not applicable.
